# Human Wharton’s jelly mesenchymal stem cells protect neural cells from oxidative stress through paracrine mechanisms

**DOI:** 10.2144/fsoa-2020-0036

**Published:** 2020-09-17

**Authors:** Teresa Puig-Pijuan, Mariana A de Godoy, Luiza Rachel Pinheiro Carvalho, Victor Bodart-Santos, Rafael Soares Lindoso, Pedro Moreno Pimentel-Coelho, Rosalia Mendez-Otero

**Affiliations:** 1 Instituto de Biofísica Carlos Chagas Filho, Universidade Federal do Rio de Janeiro, Rio de Janeiro, Brazil; 2 Instituto Nacional de Ciência e Tecnologia em Medicina Regenerativa, Rio de Janeiro, Brazil; 3 Centro Nacional de Biologia Estrutural e Bioimagem, Universidade Federal do Rio de Janeiro, Rio de Janeiro, Brazil

**Keywords:** cell therapy, extracellular vesicles, medicinal signaling cells, mesenchymal stem cells, oxidative stress, paracrine signaling, umbilical cord Wharton’s jelly

## Abstract

**Aim:**

Mesenchymal stem cells (MSCs) have neuroprotective and immunomodulatory properties, which are partly mediated by extracellular vesicles (EVs) secretion. We aimed to evaluate the effects of human Wharton’s jelly-derived MSCs (WJ-MSCs) and their EVs on rat hippocampal cultures subjected to hydrogen peroxide (H_2_O_2_).

**Materials & methods:**

Hippocampal dissociated cultures were either co-cultured with WJ-MSCs or treated with their EVs prior to H_2_O_2_ exposure and reactive oxygen species levels and cell viability were evaluated.

**Results:**

Coculture with WJ-MSCs or pre-incubation with EVs prior to the insult reduced reactive oxygen species after H_2_O_2_ exposure. Cell viability was improved only when coculture was maintained following the insult, while EVs did not significantly improve cell viability.

**Conclusion:**

WJ-MSCs have potential antioxidant and neuroprotective effects on hippocampal cultures which might be partially mediated by EVs.

## Lay abstract

Mesenchymal stem cells (MSCs) have emerged as a promising therapeutic approach for neurodegenerative diseases, yet their potential and mechanism of action have not been fully described. In this work we show that Wharton’s jelly-derived MSCs (WJ-MSCs) obtained from the human umbilical cord release vesicles that were able to reduce oxidative stress in neural cell cultures exposed to hydrogen peroxide, a potent oxidant agent. WJ-MSCs were also able to protect neural cells from hydrogen peroxide-induced cell death when they were present in the damaging environment, while treatment with WJ-MSC-derived vesicles alone did not prevent cell death at the doses used.

Reactive oxygen species (ROS) are constantly produced in the cells as a byproduct of metabolic activity. An excess of ROS production, however, can induce oxidative damage to proteins, lipids and DNA, eventually leading to cell death. Oxidative stress is a common feature in pathological conditions, including most neurodegenerative diseases [1]. The CNS is especially sensitive to oxidative damage given its high oxygen uptake and lipid content. Neurons from the hippocampus and substantia nigra, for example, are particularly vulnerable to oxidative stress [2] and, for this reason, regulation of ROS production and scavenging is a promising therapeutic target for neurodegenerative diseases that affect these brain regions, including Alzheimer’s and Parkinson's diseases.

In recent years, mesenchymal stem cells (MSCs) have been increasingly studied due to their trophic actions and immunomodulatory properties, which have been shown to promote tissue repair after different types of damage [3,4]. In particular, Wharton’s jelly-derived MSCs (WJ-MSCs) from the umbilical cord are especially interesting candidates for application in cell therapies [5–8]. Compared with MSCs from adult tissues, WJ-MSCs have differential immunoregulatory and proliferative capacities, possibly due to their early origin [9,10]. In addition, WJ-MSCs have an easy and painless collection procedure, since they can be extracted by noninvasive methods from a tissue that is usually discarded.

Several groups have demonstrated the neuroprotective and regenerative potential of MSCs in the nervous system [11–14] and numerous studies have shown that WJ-MSCs are able to improve neurological function in models of ischemic stroke, traumatic brain injury, neonatal hypoxia-ischemia and Alzheimer’s disease, among others [15–18], possibly through the release of trophic and immunoregulatory factors [16,19]. These data have led to the initiation of clinical trials for the treatment of neurodevelopmental and neurodegenerative diseases with WJ-MSC [5].

Multiple lines of evidence support the hypothesis that the beneficial effects of MSCs are mediated by their paracrine action rather than by the replacement of damaged cells [20]. Secretion of extracellular vesicles (EVs) carrying different types of biomolecules has been identified as an important form of paracrine signaling by MSCs [21]. EVs act as intercellular signaling particles through several mechanisms, including the transfer of proteins, lipids, mRNA, miRNA and transcription factors to other cells [22]. For instance, Lindoso *et al.* showed that miRNAs transferred from MSCs-derived EVs exerted changes in the gene expression of injured renal cells [23]. Administration of MSCs-derived EVs, therefore, has emerged as an alternative to cell transplantation and has been tested in different *in vivo* models of brain injury [24–27]. The utilization of *in vitro* models, on the other hand, has proven to be important for the investigation of how MSCs and their EVs exert therapeutic actions in neural cells. Recently, it was shown that EVs from rat bone marrow MSCs protected hippocampal neurons from oxidative stress induced by soluble oligomers of the amyloid-β peptide and that catalase activity in EVs was necessary for this effect [11]. Moreover, Joerger-Messerli *et al.* demonstrated that WJ-MSCs-derived EVs protected Neuro2a cells, a mouse neuroblastoma cell line, from oxygen–glucose deprivation/reoxygenation-induced cell death [28]. The neuroprotective action of human WJ-MSCs-derived EVs against oxidative stress, however, is less known.

Here, we show that human WJ-MSCs and EVs secreted by these cells reduce intracellular ROS in hippocampal cultures submitted to oxidative stress-mediated by hydrogen peroxide (H_2_O_2_). We also show that while WJ-MSCs are able to protect hippocampal cells from H_2_O_2_-induced cell death, EVs derived from WJ-MSCs do not improve cell survival.

## Materials & methods

### Cell cultures

Cell culture reagents were purchased from Thermo Fisher Scientific (Waltham, MA, USA) unless specified otherwise. Pregnant Wistar rats were used in this study. All animals received humane care in compliance with the ‘Principles of Laboratory Animal Care’ formulated by the National Society for Medical Research and the US National Academy of Sciences Guide for the Care and Use of Laboratory Animals. The procedures were approved by the Ethics Committee for Animal Experimentation of the Federal University of Rio de Janeiro (Protocol Number 055/15).

Primary hippocampal cultures were obtained from rat embryos at gestational age E17–E19. Briefly, brains were removed from the embryos and the hippocampi were carefully dissected. Hippocampal tissue was digested with trypsin for 15 min and centrifuged at 300 *g* for 5 min. The resulting cells were resuspended in Neurobasal Medium supplemented with 2% B27 Supplement, 1% GlutaMax, 1% penicillin/streptomycin (P/S) and 1% Fungizone and plated on 24-well plates containing coverslips coated with 100 μg/ml poly-L-lysine (P1524; Sigma-Aldrich, St Louis, MO, USA) and 5 μg/ml laminin. Cells were maintained in standard culture conditions (37°C, 5% CO_2_) for 13 days.

Umbilical cords were collected after informed consent forms were signed by the mothers. The Wharton’s jelly was dissected from the rest of the tissue and digested with 200 μg/ml type II collagenase for 16 h under constant shaking. Digested tissue was centrifuged at 2000, 1000 and 500 *g* for 15 min per cycle and the pellet was resuspended in phosphate-buffered saline (PBS) before each centrifugation. Cells were plated in 75 cm^2^ flasks containing DMEM/F-12 medium supplemented with 10% fetal bovine serum (FBS) and 1% P/S and were maintained at 37°C and 5% CO_2_. For the immunophenotypic characterization of these cells, see [29].

### Immunocytochemistry for neural markers

Cells grown on coverslips were fixed in PBS with 4% paraformaldehyde for 15 min and then washed and permeabilized with 0.1% Triton X-100 for 5 min. Blocking was performed by incubation in PBS with 10% Normal Goat Serum for 1 h and coverslips were incubated overnight at 4°C with primary antibodies (1:100 anti-Tuj1 MAB1637, Millipore, Burlington, MA, USA; and 1:1000, GFAP AB7260, Abcam, Cambridge, UK). Coverslips were washed three-times with PBS and incubated with secondary antibodies (1:1000 Alexa-488 goat anti-mouse IgG A11017, Invitrogen, Waltham, MA, USA; and 1:1000 Cy3 goat anti-rabbit IgG 111-166-075, Jackson Immunoresearch, West Grove, PA, USA) and diluted in blocking solution for 2 h at room temperature. Cells were stained with Hoechst 33342 for 5 min, washed and mounted on glass slides. Fluorescence images were acquired using an inverted microscope equipped with a digital camera (Zeiss Axiovert 200M, Oberkochen, Germany).

### Establishment of the co-culture system

For the coculture model, WJ-MSCs in passage 3–6 from four different donors were plated in Millicell Hanging Cell Culture Inserts (PIRP12R48, Merck Millipore) at a density of 3500 cells per insert and maintained in DMEM/F-12 culture medium supplemented with 10% FBS and 1% P/S for 60 h. Before starting the coculture, WJ-MSCs in the inserts were maintained in Neurobasal Medium supplemented with 2% B27 Supplement, 1% GlutaMax, 1% P/S and 1% Fungizone for 24 h to allow the cells to adapt to the new environment. The inserts were then transferred to wells containing 13-day hippocampal cultures. Coculture was maintained for 24 h before starting the experiments.

### Extracellular vesicles isolation & treatment

WJ-MSCs were plated in 150 cm^2^ flasks and maintained in culture until confluence was reached. Cells were washed and then maintained in FBS-free DMEM/F12 culture medium supplemented with 1% P/S for 24 h. The medium was collected and centrifuged at 2000 *g* for 20 min at 4°C. Meanwhile, cells were counted in order to calculate the production of EVs per cell. The supernatant was centrifuged at 100,000 *g* for 2 h at 4°C and the resulting EV pellet was resuspended in PBS with 1% DMSO (D2650, Sigma). Size and concentration of EV preparations were determined using the NanoSight NS300 Instrument (Malvern Instruments, Malvern, UK). With these data, we observed that each WJ-MSC produced around 2.67 × 10^4^ EVs per cell in 24 h in our culture condition. In this work, we decided to use a concentration of EVs that would be equivalent to the amount of EVs that WJ-MSCs in the co-culture system would produce in 24 h (1×) and three-times that amount (3×). On the day of the H_2_O_2_ insult, we counted a total of 21,171 ± 1,592 WJ-MSCs on the Millicell membrane, which corresponds to a total of (2.12 × 10^4^) × (2.67 × 10^4^) = 5.67 × 10^8^ EVs released by these cells in 24 h. Accordingly, hippocampal cultures were incubated with this amount of EVs 24 h prior to the 5 mM H_2_O_2_ insult, maintaining the same parameters described for the coculture with WJ-MSC. Experimental condition testing a higher amount of EVs (1.7 × 10^9^ EVs; 3× the equivalent production by WJ-MSC) was also added to the experiments. For comparison, the amount of EVs used in the experimental conditions can also be expressed as 3.40 × 10^8^ EVs/ml (1×) or 1.02 × 10^9^ EVs/ml (3×), according to the volume of medium in the wells.

### ROS detection

After 24 h in the coculture system or 24 h after being pre-incubated or not with WJ-MSC-derived EVs, the conditioned medium was collected, Millicell inserts were removed and neural cells were incubated with 2 μM CM-H2DCFDA probe (C6827, Thermo Fisher Scientific) for 30 min. After probe internalization, cells were washed once with PBS. In the coculture experiments, the Millicell inserts and the conditioned media (containing factors secreted by WJ-MSCs during the 24h prior to the experiment) were returned to the cultures. In the EVs-treatment experiment, conditioned media (containing EVs or vehicle incubated during the 24h prior to the experiment) were returned to the cultures. Neural cells were then incubated with 5 mM H_2_O_2_ for 20 min and images were acquired immediately, using an inverted microscope equipped with a digital camera (Zeiss Axiovert 200M). For each experiment, 5 images were captured on each coverslip. Mean fluorescence intensity per coverslip was quantified with ImageJ software. In order to compare ROS levels between groups, fluorescence intensity values were normalized to the control group.

### Cell viability assay in neural cultures & cocultured WJ-MSCs

After 24 h in the coculture or 24 h after being pre-incubated or not with WJ-MSC-derived EVs, half of the conditioned medium was collected. Cultures were incubated with 5 mM H_2_O_2_ for 20 min, washed twice and then returned to the previously collected conditioned medium. At this point, in one experiment Millicell inserts were removed from the wells, while in the other, Millicell inserts remained in the wells. Cells were maintained in standard culture conditions for 24 h, gently washed once and incubated with 2 μM calcein AM and 2 μM ethidium homodimer-1 from the LIVE/DEAD Viability/Cytotoxicity Kit (L3224, Invitrogen) for 10 min. Cells were then washed once and images were immediately acquired, using an inverted microscope equipped with a digital camera (Zeiss Axiovert 200 M).

After the second 24h coculture phase, WJ-MSCs viability was also assessed by incubating the Millicell Inserts with 2 μM calcein AM and 2 μM ethidium homodimer-1 (LIVE/DEAD Viability/Cytotoxicity Kit L3224, Invitrogen) for 10 min. Inserts were washed once with PBS, fixed with 4% paraformaldehyde, gently washed three-times with PBS and stained with Hoechst 33342 for nuclei visualization. After two more washes, the membranes were cut from the inserts and mounted on glass slides. Fluorescence images were acquired using a Zeiss Axiovert 200M microscope equipped with a digital camera. Viable and apoptotic cells were quantified using ImageJ software.

### Statistical analysis

All statistical analyses were performed using Prism software (Version 6.01; GraphPad Software Inc.). For the coculture model, two-way analysis of variance (ANOVA) followed by Tukey’s test was used. For the experiments with EVs, one-way ANOVA followed by Tukey’s test was used. Cell viability in WJ-MSCs was analyzed using Student’s t-test. The observed differences were considered significant when p < 0.05.

## Results

### Characterization of primary hippocampal cell cultures

**Figure 1.**
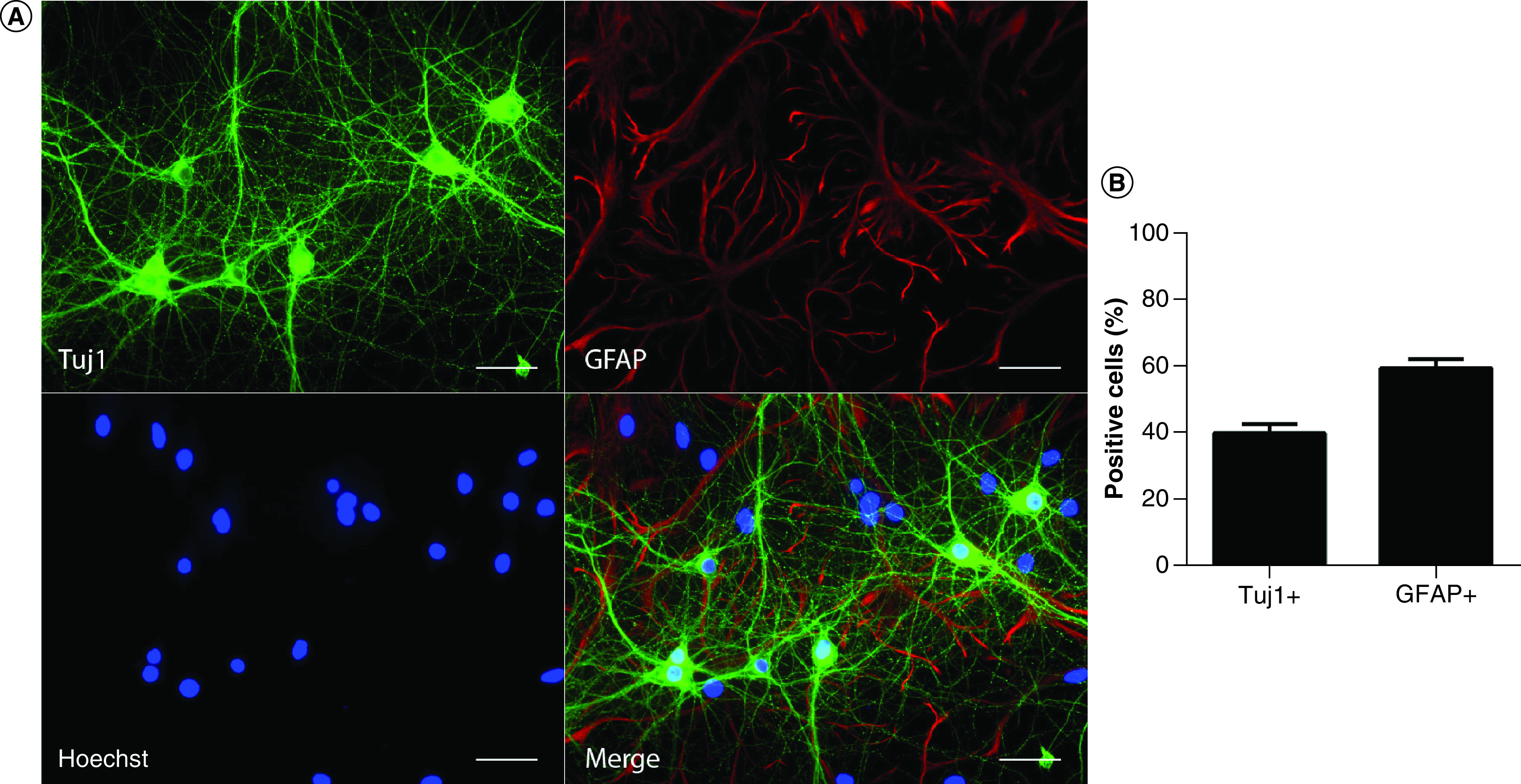
Immunofluorescence analysis of 14-day neural cell cultures from E17–E19 rat hippocampus. **(A)** Cells were labeled with the neuronal marker Tuj1 (green) and the astrocyte marker GFAP (red). Nuclei were stained with Hoechst (blue). Scale bars: 50 μm. **(B)** Percentage of neurons (Tuj1+) and astrocytes (GFAP+) in the hippocampal cell cultures. Bars indicate mean ± standard error. Each dataset was calculated from n = 6.

In this study, primary hippocampal cultures from rat E17–E19 embryos were grown in standard culture conditions for 13 days before experiments were started. In order to evaluate the cellular composition of our cultures on the day of the experiments, immunocytochemistry for typical markers of neurons (Tuj1: β-tubulin class III) and astrocytes (GFAP) was performed. Tuj1 staining indicated that 14-day cultured neurons had a highly ramified dendritic tree. The presence of a “feeder layer” of astrocytes was shown by GFAP staining (Figure 1A). Quantification of Tuj1+ cells and GFAP+ cells revealed that our primary hippocampal cultures consisted of 40.2 ± 2.8% neuronal cells and 59.8 ± 2.8% astrocytes (Figure 1B).

### WJ-MSCs reduce ROS levels in neural cells after induction of oxidative stress with hydrogen peroxide

**Figure 2.**
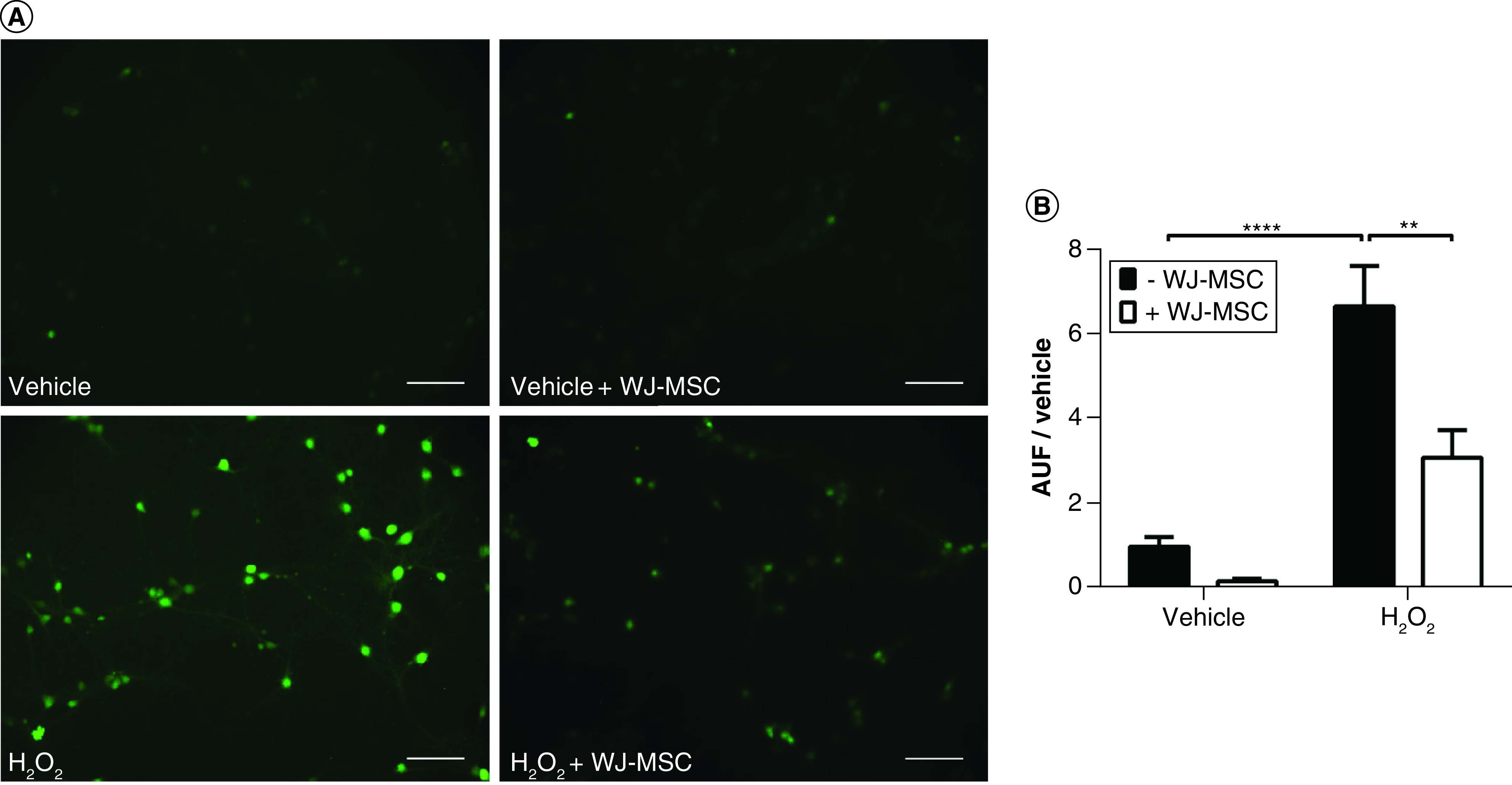
Wharton’s jelly-derived mesenchymal stem cells reduce reactive oxygen species levels in neural cells after induction of oxidative stress with hydrogen peroxide. **(A)** Reactive oxygen species were detected in neural cells using the CM-H2DCFDA probe, which emits green fluorescence. Scale bars: 100 μm. **(B)** Quantification of the fluorescence intensity of the reactive oxygen species-sensitive CM-H2DCFDA. Results are displayed as arbitrary units of fluorescence divided by the control (vehicle treated) value (AUF/control). Each n represents the mean fluorescence intensity of five random fields from each coverslip (n = 9–11), from a total of four independent experiments. Bars illustrate mean ± SE. **p < 0.01; ****p < 0.0001. Statistical analysis: two-way ANOVA followed by Tukey’s test. AUF: Arbitrary units of fluorescence; H_2_O_2_: Hydrogen peroxide; WJ-MSC: Wharton’s jelly-derived mesenchymal stem cell.

One of the goals of our study was to evaluate the capacity of WJ-MSCs to modulate intracellular ROS levels in neural cells undergoing oxidative stress. For this purpose, WJ-MSCs were plated in transwell membranes and cocultured with 13-day neural cell cultures for 24 h and then oxidative stress was triggered by incubation with 5 mM H_2_O_2_ for 20 min. The CM-H2DCFDA probe was used to detect changes in intracellular ROS levels. As shown in Figure 2A and B, H_2_O_2_ treatment significantly increased ROS levels in hippocampal cells (6.7 ± 0.8 AUF [arbitrary units of fluorescence]; p < 0.0001). However, the presence of WJ-MSC in the coculture system significantly reduced ROS levels in hippocampal cells by more than 50% (3.1 ± 0.9 AUF; p = 0.0011). Although there was a reduction of basal ROS levels in vehicle-treated cells cocultured with WJ-MSCs compared with vehicle-treated cells in the absence of WJ-MSCs, this reduction was not statistically significant (0.2 ± 0.8 AUF; p = 0.77). This result demonstrates that the presence of WJ-MSC in coculture is capable of reducing ROS levels in neural cells exposed to H_2_O_2_.

### WJ-MSCs improve cell viability after induction of oxidative stress through cell-dependent mechanisms

Considering that pretreatment with WJ-MSCs in a coculture system prevented the generation of ROS in neural cells exposed to hydrogen peroxide, we next aimed to evaluate the ability of WJ-MSCs to protect neural cells from cell death.

**Figure 3.**
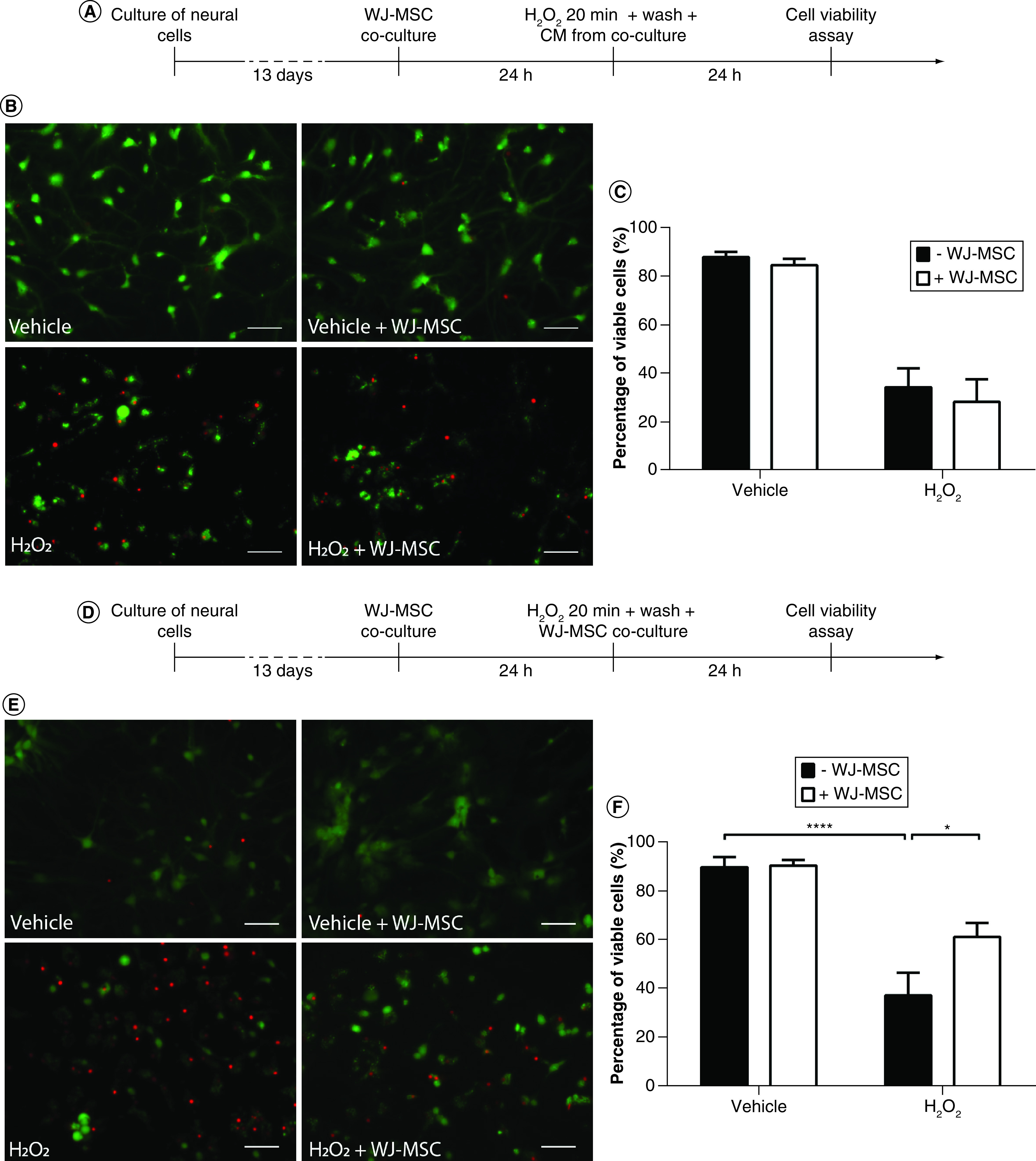
The presence of Wharton’s jelly-derived mesenchymal stem cells after the H_2_O_2_ insult is necessary for their neuroprotective effect. Neural cells were either pre-incubated with WJ-MSCs and incubated with WJ-MSC-derived conditioned medium (CM) after the H_2_O_2_insult **(A–C)**, or cocultured with WJ-MSCs before and after the H_2_O_2_ insult **(D–F)**. **(A & D)** Experimental design. **(B & E)** Representative images of cells stained with the LIVE/DEAD kit. Viable cells were stained with calcein-AM (green) while dead cells were stained with ethidium homodimer-1 (red). Scale bars: 50 μm. **(C & F)** Quantification of cell viability, expressed as the percentage of viable cells. Each n represents the mean fluorescence of four random fields per coverslip, from a total of two independent experiments (n = 5–6 for each group). Bars illustrate mean ± SE. CM: Conditioned medium; H_2_O_2_: Hydrogen peroxide; WJ-MSC: Wharton’s jelly-derived mesenchymal stem cell.

In the first experiment (Figure 3A), neural cells were pre-incubated or not with WJ-MSCs for 24 h in a transwell coculture system and then oxidative stress was induced by adding 5 mM H_2_O_2_ to the culture medium. After 20 min, H_2_O_2_ was removed by washing the culture with PBS. Then, neural cells that had been pre-incubated with WJ-MSCs were maintained for an additional 24 h with conditioned medium from the coculture but without WJ-MSCs. In order to assess cell viability, the LIVE/DEAD assay was performed. We found that neural cells treated with vehicle had basal viability of 88.0 ± 2.1% (Figure 3B & C). Pre-incubation with WJ-MSCs did not significantly improve the viability of vehicle-treated cells not exposed to H_2_O_2_ (84.6 ± 2.6% viable cells; Figure 3B & C). H_2_O_2_ significantly decreased the survival of neural cells (34.5 ± 7.8% viable cells) and this did not change when the cells were pre-incubated with WJ-MSCs before and during the insult (28.4 ± 9.3% viable cells; Figure 3B & C). These results suggest that although the presence of WJ-MSCs before and during the H_2_O_2_ insult decreases ROS levels in neural cells, the remaining ROS levels are sufficient to trigger oxidative stress and lead to cell death within the next 24 h.

Then, we asked whether it would be possible to prevent the death of neural cells by maintaining them in coculture with WJ-MSC for another 24 h after incubation with H_2_O_2_ (Figure 3D). Under basal conditions, viability rates were very similar to those observed in the previous experiment: 89.9 ± 4.2% in the vehicle group and 90.6 ± 2.3% in the vehicle + WJ-MSC group. Interestingly, maintaining WJ-MSC cocultures for an additional 24 h following incubation with H_2_O_2_ significantly increased the viability of neural cells (37.6 ± 9.2% viable cells in the H_2_O_2_ group vs 61.5 ± 5.7% in the H_2_O_2_ + WJ-MSC group; Figure 3E & F). These results show that the presence of WJ-MSC after oxidative stress induction is crucial for neuroprotection.

**Figure 4.**
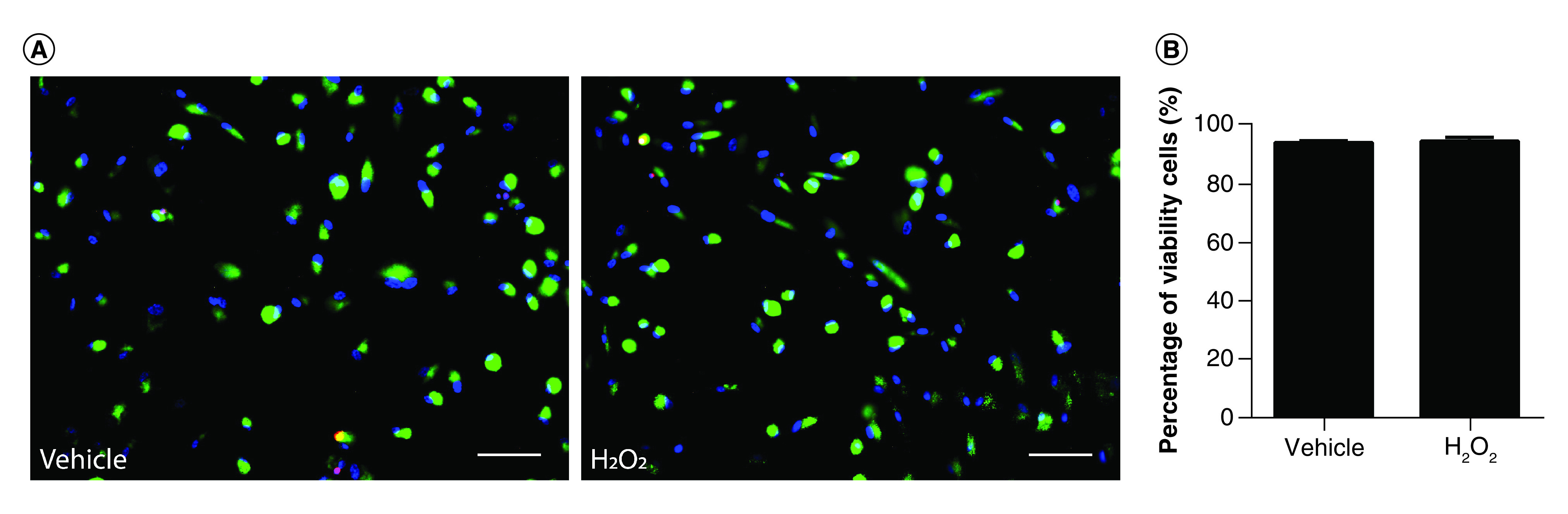
Wharton’s jelly-derived mesenchymal stem cells in coculture do not show changes in cell viability 24 h after H_2_O_2_ exposure. **(A)** Wharton’s jelly-derived mesenchymal stem cells adhered on Millicell Inserts were stained with the LIVE/DEAD kit 24 h after the H_2_O_2_ insult. Viable cells were stained with calcein-AM (green) while dead cells were stained with ethidium homodimer-1 (red). Scale bars: 100 μm. **(B)** Quantification of cell viability, expressed as the percentage of viable cells. No significant differences in cell viability were observed between vehicle and H_2_O_2_ groups. Bars illustrate mean ± standard error, n = 6. Statistical analysis: Student’s t-test. H_2_O_2_: Hydrogen peroxide.

In order to provide antioxidant neuroprotection, WJ-MSCs must be able to efficiently manage ROS and resist their toxic effects. Therefore, in addition to studying the effect of WJ-MSCs on hippocampal cells, we analyzed the viability of WJ-MSCs from the Millicell inserts exposed to H_2_O_2_. WJ-MSCs from Millicell membranes that were maintained in coculture for 24 h following incubation with H_2_O_2_ were stained with the LIVE/DEAD kit and fixed immediately. Quantification of cell viability showed no significant differences between vehicle-treated WJ-MSCs and H_2_O_2_-treated WJ-MSCs (94.8 ± 0.6% vs 95.4 ± 1.3%, respectively) (Figure 4A & B), demonstrating that WJ-MSCs are resistant to oxidative stress.

### WJ-MSC-EVs reduce ROS levels in neural cells submitted to oxidative stress

In our coculture system, communication between WJ-MSCs and neural cells occurs in a paracrine manner, since the membrane pores only allow the passage of molecules and particles up to 1 μm. One of the most important forms of paracrine signaling is the release of EVs, which may be involved in the protective effects of WJ-MSCs observed in our model, considering their ability to pass through Millicell membranes.

For EV collection, WJ-MSCs were deprived of fetal bovine serum for 24 h and the culture medium was collected and subjected to ultracentrifugation. Size and concentration of the particles in our samples were analyzed using the NanoSight NS300 Instrument, showing a predominance of EVs measuring 70–250 nm, with a peak around 100 nm (Supplementary Figure 1).

**Figure 5.**
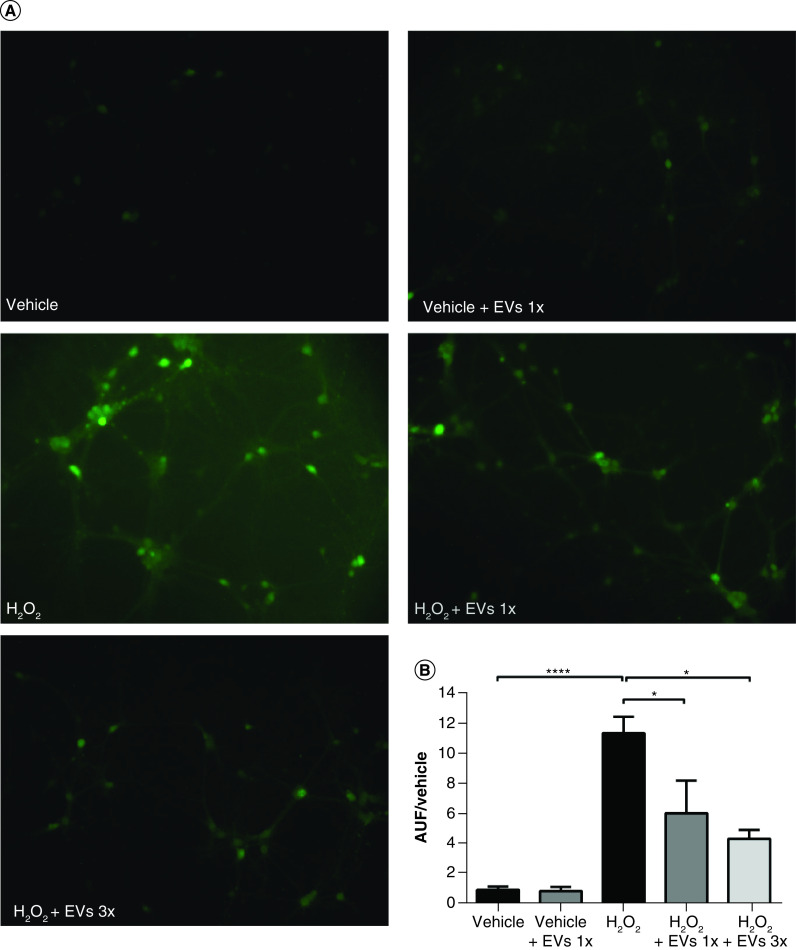
Pretreatment with Wharton’s jelly-derived mesenchymal stem cell-derived extracellular vesicles reduces reactive oxygen species levels in neural cells exposed to H_2_O_2_. **(A)** Representative images of ROS detection by CM-H2DCFDA probe in neural cells exposed to H_2_O_2_ or vehicle. Cells were pre-incubated or not with EVs for 24 h before the insult. Scale bars: 100 μm. **(B)** Quantification of fluorescence intensity of the ROS-sensitive probe CM-H2DCFDA, expressed in AUF divided by the control group (vehicle). Each n represents the mean fluorescence intensity of five random fields per coverslip (n = 5, except n = 3 in the H_2_O_2_ + EVs 3× group), from a total of two independent experiments. Bars illustrate mean ± standard error. ****p < 0.0001; *p < 0.05. Statistical analysis: one-way ANOVA followed by Tukey’s test. AUF: Arbitrary units of fluorescence; EV: Extracellular vesicle; H_2_O_2_: Hydrogen peroxide.

We first investigated the ability of WJ-MSC-derived EVs to reduce ROS levels in hippocampal cultures exposed to H_2_O_2_. We analyzed the levels of intracellular ROS in neural cells treated with EVs for 24 h and subsequently incubated with H_2_O_2_ for 20 min (Figure 5A). The dose of EVs used was chosen by estimating the amount of EVs that WJ-MSCs in the Millicell inserts would produce during the 24 h of coculture (5.67 × 10^8^ VEs, described as 1×) and a dose three-times higher (3×). This analysis showed a significant increase in ROS levels in the presence of H_2_O_2_ (1.0 ± 0.2 AUF in the vehicle vs 11.5 ± 1.1 AUF in the H_2_O_2_ group; p < 0.0001) (Figure 5B). However, when hippocampal cells were pre-incubated with EVs (1×) for 24 h, ROS levels were significantly reduced by almost half compared with the group without pre-incubation with EVs (6.1 ± 2.2 AUF; p = 0.032). With a higher concentration of EVs (3×), the reduction of ROS levels was slightly higher (4.4 ± 0.6 AUF; p = 0.012). These results show that EVs from WJ-MSCs have antioxidant potential in hippocampal cells.

### WJ-MSC-EVs do not reduce cell death in neural cells submitted to oxidative stress

We also analyzed the capability of WJ-MSC-derived EVs to protect neural cells exposed to H_2_O_2_ from cell death. After 13 days in culture, hippocampal cells were incubated with the vehicle, EVs 1× or EVs 3× for 24 h and then treated with 5 mM H_2_O_2_ for 20 min. The cells were maintained for 24 h in the conditioned medium and then the LIVE/DEAD viability assay was performed.

**Figure 6.**
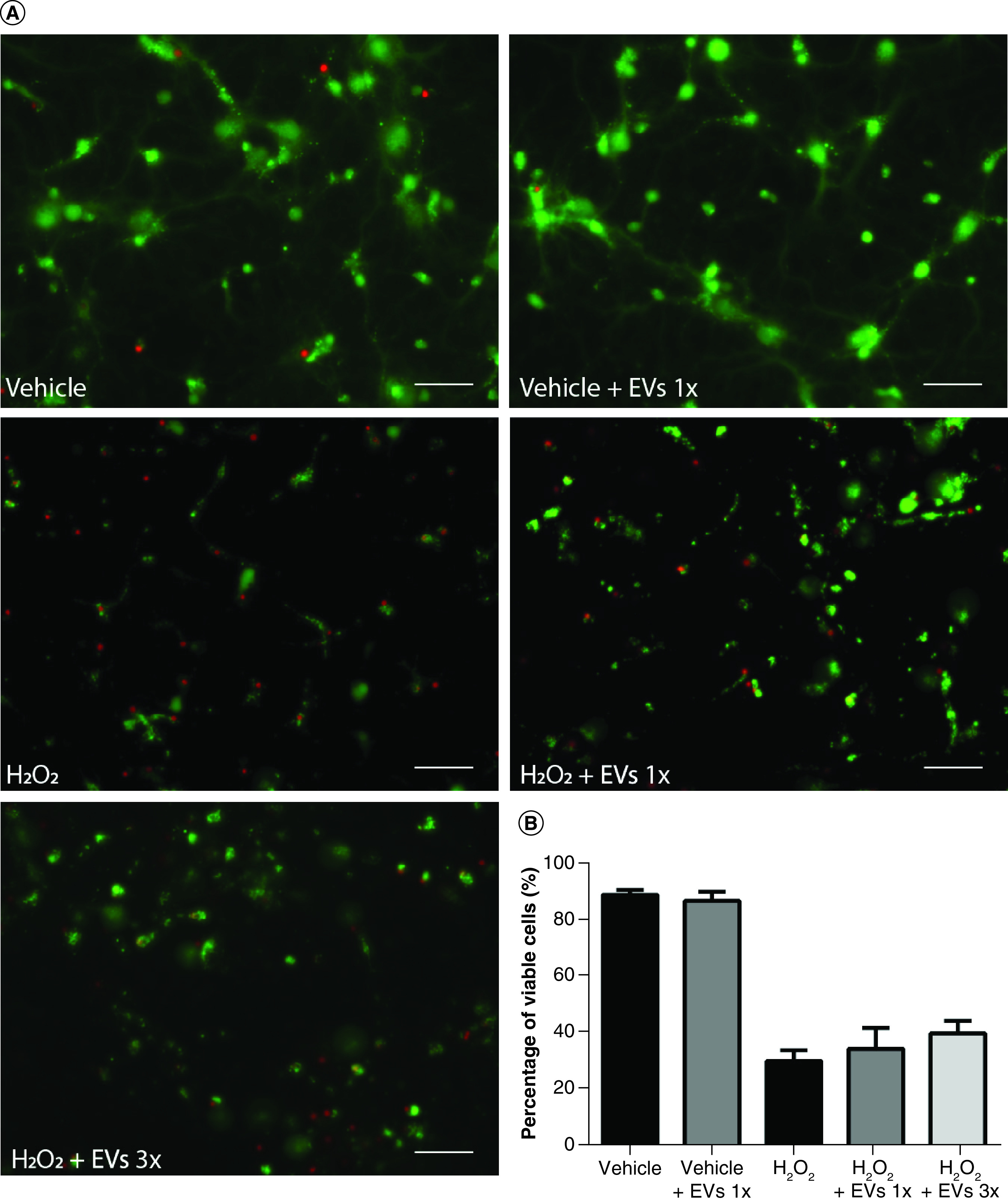
Pretreatment with Wharton’s jelly-derived mesenchymal stem cell-derived extracellular vesicles does not significantly increase cell viability after H_2_O_2_ exposure. **(A)** Representative images of cells stained with the LIVE/DEAD kit 24 h after the H_2_O_2_ insult. Viable cells were stained with calcein-AM (green), while dead cells were stained with ethidium homodimer-1 (red). Scale bars: 50 μm. **(B)** Quantification of cell viability expressed as the percentage of viable cells, obtained from five independent experiments (n = 9–10). Bars illustrate mean ± standard error. ****p < 0.0001. Statistical analysis: one-way ANOVA followed by Tukey’s test. AUF: Arbitrary units of fluorescence; EV: Extracellular vesicle; H_2_O_2_: Hydrogen peroxide.

Again, the results showed a decrease in viability when neural cells were exposed to H_2_O_2_ (89.3 ± 1.8% viable cells in the vehicle vs 30.3 ± 3.8% in the H_2_O_2_-treated group, p < 0.0001). When hippocampal cells were treated with EVs before the H_2_O_2_ insult, we observed a slight but nonsignificant improvement in cellular viability (Figure 6). In summary, these data show that although EVs are capable of reducing intracellular ROS in neural cells, this reduction is not sufficient to prevent neural death induced by H_2_O_2_.

## Discussion

In this work, we investigated the capacity of WJ-MSCs to protect neural cells from oxidative stress caused by H_2_O_2_. The ability of WJ-MSC-derived EVs to reproduce the effects of cellular therapy was also studied. WJ-MSC transplantation has previously shown therapeutic effects in disease models where oxidative stress occurs, including acute kidney injury [30], stroke [19] and Alzheimer’s disease [17,31], among others. However, these studies have not demonstrated whether WJ-MSCs directly regulate ROS levels, or if oxidative stress is reduced as a consequence of an improvement in the pathology (i.e, improvement of kidney function in acute kidney injury or amyloid-β clearance in Alzheimer's disease) mediated by WJ-MSCs. In this study, we show for the first time that WJ-MSCs and their EVs have a direct impact on intracellular ROS levels in neural cells exposed to an oxidizing agent.

It is now clear that the therapeutic potential of MSCs is not limited to the replacement of damaged cells, but most likely involves paracrine mechanisms for tissue regeneration. WJ-MSCs release neuroprotective and growth factors that influence nearby and distant cells, creating a favorable microenvironment that promotes tissue regeneration and repair [20]. We propose that the protective effect of WJ-MSCs that we observed was mediated by paracrine signaling, mediated at least in part by EVs release, since WJ-MSCs and neural cells were separated by a transwell membrane. SOD1, SOD2, catalase and GPx are crucial enzymes for cellular ROS managing and it has already been shown that they are expressed and active in WJ-MSCs [32]. The secretome of WJ-MSCs in coculture potentially contain these antioxidant enzymes or mRNAs for these enzymes, which can be translated once incorporated by neural cells. In fact, a recent study of our group registered catalase activity in EVs from WJ-MSCs and observed that these EVs were internalized by neural cells [33], a mechanism previously described by others [11,34]. Besides the transfer of proteins or specific mRNAs that code for redox enzymes, the transfer of miRNAs that act on cellular pathways in target cells has also been described as one of the therapeutic effects of MSCs [35,36]. Although this work focused on the paracrine actions of WJ-MSC, it is important to mention that these cells can also have other therapeutic effects mediated by cell-to-cell contact [37], which could not be considered here since we used a transwell-based coculture design.

Our study focused on studying the effect that WJ-MSCs could have in protecting neural cell viability from an oxidant environment, considering that oxidative stress-induced cell death is a common feature in many neurological disorders [38]. For this reason, we used a strong H_2_O_2_ stimulus that caused extensive cell death of neural cells within 24 h. Since we have used such a damaging environment, studying other aspects of neural cell function, such as synaptic functioning, was considered out of the scope of this work. Here, while pre-incubation with WJ-MSC did not improve cell death, WJ-MSCs were able to preserve cell viability when the coculture was maintained for 24 h after the H_2_O_2_ insult. These results show that the presence of WJ-MSCs after the insult is crucial for the improvement of hippocampal cell survival. Importantly, many studies that have assessed the paracrine effects of MSCs have transferred the conditioned medium from MSCs to other cell cultures [39,40]. In the present study, we used a coculture system that allowed the bi-directional interaction between WJ-MSCs and neural cells, without physical contact between the two cell types. This is a more dynamic system that reproduces how the secretome of MSCs can be affected by the dying cells and, in turn, the MSCs can provide neuroprotection in a paracrine fashion. Previous studies have shown that environmental conditions such as hypoxia and inflammation can change the gene expression profile of MSCs and modify the protein content and functional properties of their EVs [41,42]. Moreover, it has been observed that MSCs preconditioned with H_2_O_2_ display changes in their gene expression [43,44], which can improve some of the therapeutic effects of WJ-MSCs [45]. Taking this into consideration, we hypothesize that the presence of H_2_O_2_ in the medium, as well as of danger signals produced by stressed neurons and astrocytes, might have triggered WJ-MSCs to release more trophic factors and/or EVs containing mRNAs, miRNAs and proteins that protected neural cells against oxidative stress and cell death. These triggering stimuli did not occur in our EV treatment assay, since EVs were extracted from unprimed WJ-MSCs, so it could explain why we did not observe significant changes in the viability of neural cells treated with EVs, even at a higher dose. Another reason for the lack of efficacy of EVs in cell survival could be that neuroprotection from cell death was mediated by molecules that were not transferred by secreted EVs. To our knowledge, two studies have shown that murine adipose-derived stromal cells vesicles can protect neuronal cells from H2O2-induced cell death [46,47]. Here, we used human MSCs-derived EVs, which are more clinically relevant considering that the safety and feasibility of administering MSCs-derived EVs are already being tested in clinical trials [48], including in patients with neurological diseases such as stroke (ClinicalTrials.gov identifier: NCT03384433).

We cannot exclude the possibility that the lack of neuroprotective effect of the EVs treatment that we performed could be due to an insufficient dose of EVs. Previous studies have already described dose-dependent therapeutic effects of stem cell-derived EVs in different models [49–51]. In contrast, Farinazzo *et al.* and Bonafede *et al.* [46,47] found that only lower doses of murine adipose-derived stromal cells EVs protected neural cells from oxidative stress-induced cell death. In this study, we used a concentration of EVs that simulates the amount of EVs that would be released in our WJ-MSCs coculture model. We also tested the effects of a higher dose, by increasing the number of vesicles by a factor of 3, but both doses provided comparable results. Other groups have also used cell-equivalent doses [52,53] or doses which are in the same order of magnitude as ours [54,55], while others have successfully used even higher doses in animal models [51]. One of the studies that used cell-equivalent doses observed greater therapeutic effects using MSCs than MSC-derived EVs alone *in vivo* [53]. It is possible that treatments with EVs alone would require even higher doses to produce neuroprotective effects comparable to those of cell therapies. Since treatments with EVs, in theory, offer higher safety than WJ-MSCs treatment, it would be possible to use higher doses of EVs in therapy in order to obtain better effects.

Although EVs could not improve cell viability at the doses used here, they were able to reduce intracellular ROS in neural cells, which reinforces the hypothesis that WJ-MSC-derived EVs might contain molecules with antioxidant effects such as catalase that can be transferred to neural cells. The therapeutic effect of MSC-derived EVs has already been studied in different animal models of diseases that involve oxidative damage. In previous studies, treatment with EVs decreased oxidative stress and cell death by apoptosis in *in vivo* and *in vitro* models of acute renal failure [56]. In rats submitted to myocardial ischemia-reperfusion injury, treatment with EVs reduced oxidative stress and infarct size [57]. EVs also showed therapeutic effects in the CNS [58], including the improvement in motor function, angiogenesis and neuroprotection after cerebral ischemia in animal models [27,59]. WJ-MSCs-derived EVs might also be able to protect synaptic function as seen in previous work from our group [33].

In our coculture model, we must also consider the possibility that the protection was due in part to the uptake and neutralization of H_2_O_2_ from the medium by WJ-MSCs. However, we must remember that the administration of EVs lead to a reduction in ROS levels similar to the reduction observed in our coculture, demonstrating that uptake of H_2_O_2_ was not the main antioxidant mechanism. Thus, it is clear that molecules contained in WJ-MSCs-derived EVs are capable of reducing ROS in neural cells.

## Conclusion

We demonstrated here that WJ-MSCs coculture and WJ-MSC-derived EVs reduce ROS levels in hippocampal cultures submitted to H_2_O_2_. The presence of WJ-MSCs after the insult was necessary for a significant preservation of cell viability in neural cells, which indicated that priming of WJ-MSCs might be required to promote neural cell survival. Although other studies have already shown that WJ-MSCs have beneficial effects in oxidative stress-related diseases, here we demonstrate that these cells directly combat high ROS levels. This effect is in part mediated by the release of EVs, but other EV-independent mechanisms may also be involved in the paracrine neuroprotective effect of WJ-MSCs, since WJ-MSCs-derived EVs alone reduced ROS levels but were not sufficient to restore cell survival. Altogether, these findings give a greater understanding of the real potential of WJ-MSCs and WJ-MSC-derived EVs administration as a therapeutic strategy for neurological diseases associated with oxidative stress.

## Future perspective

The therapeutic use of MSCs has been a subject of growing interest over the past 20 years and although other sources of stem cells including induced pluripotent stem cells and adipose MSC are also being studied as candidates, WJ-MSCs remain a noninvasive and readily available source of stem cells that carry less teratoma risk and ethical concerns, which makes them more interesting than cells from other origins. Clinical trials already started to evaluate the safety and efficacy of WJ-MSCs and will help us understand the true therapeutic potential of these cells, while the application of the rapid evolving ‘omics’ technologies will be crucial to unravel the molecular systems involved in the paracrine mechanisms of neuroprotection and tissue repair. As growing evidence is supporting the importance of extracellular vesicles in mediating the effects of MSC, it is important that future studies focus on optimizing the most effective doses, duration of treatments and possible preconditioning treatment of MSCs in order to obtain the best conditions for EV treatment.

**Summary points**

**Background**

- Oxidative stress plays a key role in the pathogenesis of many neurodegenerative diseases, causing neural cell death.
- Wharton’s jelly-derived mesenchymal stem cells (WJ-MSCs) possess multiple mechanisms to combat oxidative stress, including the expression of antioxidant enzymes and the release of catalase through extracellular vesicles.
- In this work, the potential of WJ-MSCs and WJ-MSC-derived extracellular vesicles (EVs) to protect neural cells from H_2_O_2_-induced oxidative stress and cell death was tested.

**Key results**

- Coculture with WJ-MSC or treatment with WJ-MSC-derived EVs significantly reduced reactive oxygen species caused by H_2_O_2_ exposure in neural cells.
- WJ-MSCs only protected neural cells from cell death when the coculture was maintained after H_2_O_2_ exposure, indicating that exposure to the oxidant agent might trigger defense mechanisms in WJ-MSCs.
- WJ-MSCs-derived EVs reduced intracellular ROS in neural cells but were not able to protect neural cells from H_2_O_2_-induced cell death.

**Conclusion**

- WJ-MSCs have antioxidant and neuroprotective properties, which make them promising therapeutic candidates in oxidative stress-related neurological diseases.
- WJ-MSC-derived EVs have an antioxidant potential, but preconditioning of MSCs or the presence of other secretome components might be necessary to fully recapitulate WJ-MSCs neuroprotective properties.
