# Supplementary figures and images for: Human Wharton’s jelly mesenchymal stem cells protect neural cells from oxidative stress through paracrine mechanisms
Source: Future Sci OA. 2020 Sep 17;6(9):FSO627. doi: 10.2144/fsoa-2020-0036 (PMC7668126; doi:10.2144/fsoa-2020-0036)

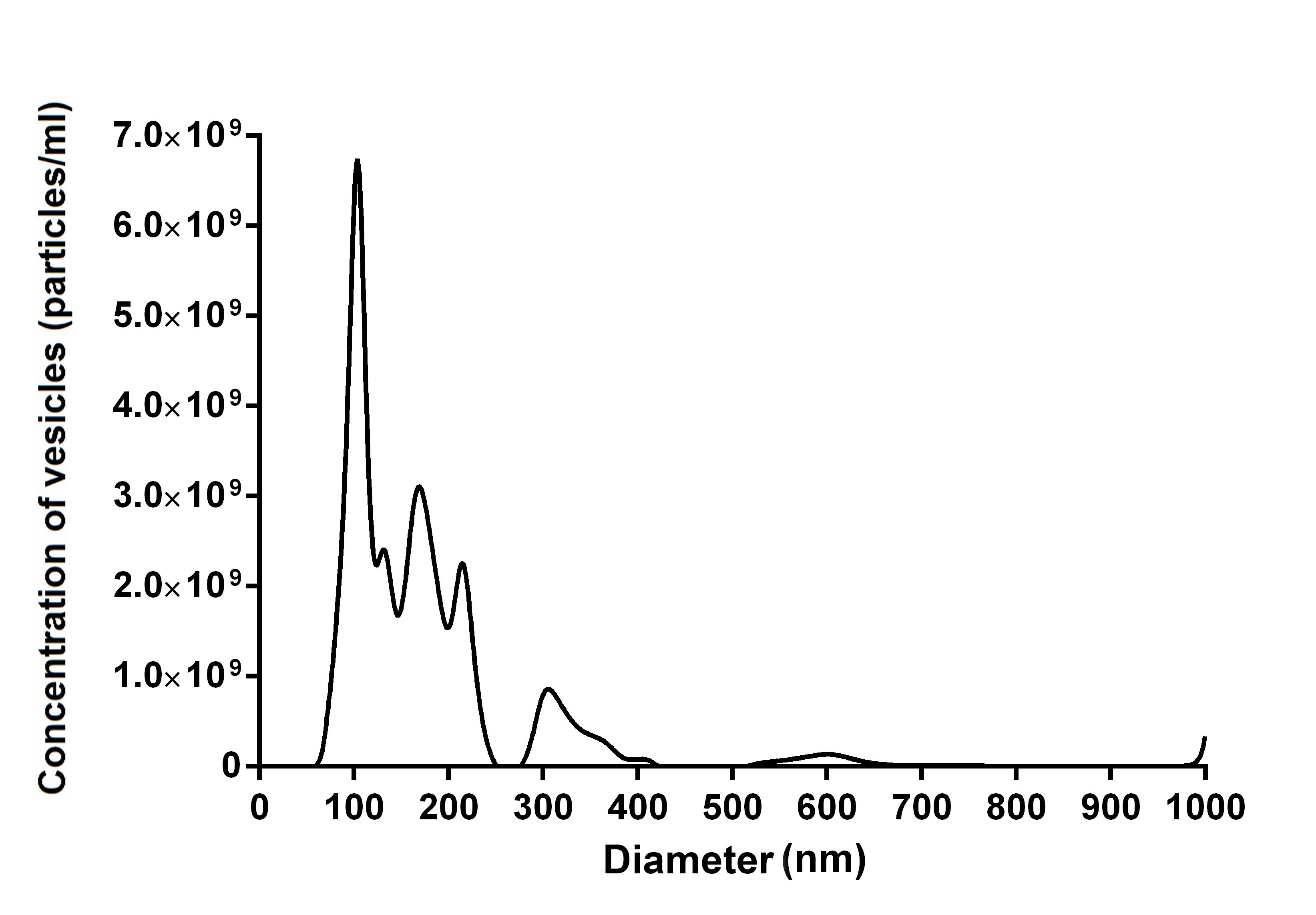

Supplement: Supplementary file 1 [file fsoa-06-627-s1.jpg]
